# Evaluating the Therapeutic Properties of Natural Products in Orthodontic and Surgical Treatment of Dentofacial Deformities: A Systematic Review of Clinical Trials

**DOI:** 10.3390/nu16121941

**Published:** 2024-06-19

**Authors:** Serban Talpos Niculescu, Robert Avramut, Tareq Hajaj, Nicoleta Nikolajevic-Stoican, Raluca Maracineanu, Antonis Perdiou, Roxana Talpos Niculescu, Marius Pricop, Roxana Ghircau-Radu, Magda Mihaela Luca, Malina Popa

**Affiliations:** 1 Discipline of Oral and Maxillo-Facial Surgery, Faculty of Dental Medicine, “Victor Babes” University of Medicine and Pharmacy Timisoara, 300041 Timisoara, Romania; talpos.serban@umft.ro; 2 Doctoral School, “Victor Babes” University of Medicine and Pharmacy Timisoara, 300041 Timisoara, Romania; robert.avramut@umft.ro (R.A.); stoican.nicoleta@umft.ro (N.N.-S.); ralucazibileanu@yahoo.com (R.M.); aperdiou@gmail.com (A.P.); pricop.marius@umft.ro (M.P.); 3 Discipline of Prostheses Technology and Dental Materials, Faculty of Dental Medicine, “Victor Babes” University of Medicine and Pharmacy Timisoara, 300041 Timisoara, Romania; tareq.hajaj@umft.ro; 4 Discipline of Odontotherapy-Endodontics, Faculty of Dental Medicine, “Victor Babes” University of Medicine and Pharmacy Timisoara, 300041 Timisoara, Romania; clinci.roxana@umft.ro; 5 Stomatology Clinic, 310025 Arad, Romania; radu.roxana50@yahoo.com; 6 Pediatric Dentistry Research Center (Pedo-Research), Department of Pediatric Dentistry, Faculty of Dental Medicine, “Victor Babes” University of Medicine and Pharmacy Timisoara, Eftimie Murgu Square 2, 300041 Timisoara, Romania; popa.malina@umft.ro

**Keywords:** systematic review, orthodontic treatment, pain management, stomatology

## Abstract

The use of natural products as alternatives to traditional pharmacological treatments in orthodontics is gaining interest due to their anti-inflammatory, antibacterial, and antioxidant properties. This systematic review synthesizes evidence from clinical trials to evaluate the efficacy of natural products in reducing inflammation and bacterial presence in orthodontic and orthognathic treatment settings. The database search was conducted across PubMed, Scopus, and Embase up to January 2024. The review focused on randomized controlled trials only. The selected studies centered on the anti-inflammatory, antibacterial, and antioxidant effects of natural products, adhering to the Preferred Reporting Items for Systematic reviews and Meta-Analyses (PRISMA) guidelines for data extraction. Nine studies, totaling 358 participants, were included. Significant findings demonstrated a reduction in gingival inflammation by over 40% with the use of Aloe vera compared to chlorhexidine. Another study noted a decrease in bleeding on probing by 13.6 points in the treatment group over placebo. Additionally, honey showed a rapid modulation of plaque pH and significantly reduced bacterial counts of *Streptococcus mutans*. Furthermore, the use of resveratrol emulgel was linked to substantial improvements in gingival health, with a reduction in the gingival index and probing pocket depth. The results indicate that natural products can significantly enhance orthodontic treatment outcomes by reducing inflammation and bacterial levels. These products offer effective alternatives to traditional treatments and show potential for integration into routine orthodontic care protocols. Further research is encouraged to standardize application methods and dosages to maximize clinical benefits and patient satisfaction.

## 1. Introduction

Orthodontic treatment, aimed at correcting irregularities in tooth alignment and jaw position, often involves the use of appliances like braces or aligners [1,2]. These interventions, while effective, can lead to biological responses such as inflammation, typically characterized by pain, swelling, and redness, which are common side effects experienced by orthodontic patients [3,4,5]. Therefore, managing these inflammatory responses is important as they can impact patient comfort and the overall effectiveness of the treatment.

Traditionally, the management of inflammation and pain during orthodontic treatment has relied on the use of non-steroidal anti-inflammatory drugs (NSAIDs) [6,7]. While NSAIDs are effective in controlling orthodontic pain and inflammation, their use is often limited by potential side effects, including gastrointestinal issues and cardiovascular risks [8,9]. Consequently, there is a growing interest in alternative treatments that are effective yet bear fewer risks, and that could be recommended for patients that have contraindications for NSAIDs [10,11,12].

Natural products have been used medicinally for thousands of years and are known for their pharmacological effects, including anti-inflammatory, anti-bacterial, and anti-oxidant properties [13,14]. These products, derived from plants, animal products, or minerals, offer a vast repository of bioactive compounds that could provide therapeutic benefits without the adverse effects associated with synthetic drugs [15,16]. The anti-inflammatory mechanisms of natural products often involve modulation of inflammation mediators such as cytokines and enzymes involved in the inflammatory pathway. Many natural extracts and compounds have been documented to inhibit these mediators, thereby reducing inflammation [17,18]. For orthodontic patients, the application of such natural agents could potentially accelerate recovery times and improve overall treatment outcomes.

Based on the growing interest and preliminary findings regarding natural products as therapeutic options, this systematic review hypothesizes that natural products are effective in reducing inflammation, provide anti-bacterial and antioxidant effect in patients undergoing orthodontic and dentofacial surgical treatment. This review aims to synthesize existing clinical trial data to provide a clear, evidence-based guide for the use of natural anti-inflammatory agents in orthodontics, potentially offering a viable alternative to traditional pharmacological treatments.

## 2. Materials and Methods

### 2.1. Protocol and Registration

For this systematic review, a comprehensive search strategy was implemented across major medical and scientific databases, including PubMed, Scopus, and Web of Science, to gather literature up to January 2024. This search was specifically designed to capture clinical trials evaluating the anti-inflammatory, antibacterial, and antioxidant effectiveness of natural products during orthodontic treatment, recognizing the variability in the types of natural products and the methods of their application in orthodontics, and differentiating studies based on the type of natural product used, the application method, and the stage of orthodontic treatment.

The search was structured around the Population-Intervention-Comparator-Outcome (PICO) framework, targeting the following criteria: orthodontic patients of any age (Population) undergoing treatment with natural products possessing anti-inflammatory properties (Intervention), compared against standard care or placebo (Comparator), to assess outcomes related to inflammation control, pain reduction, and treatment satisfaction (Outcome).

An expanded set of keywords and phrases was generated to ensure a thorough exploration of the subject. These included “Orthodontic Appliances”, “Anti-Inflammatory Agents”, Antibacterial Agents”, “Natural Products”, “Natural Remedies”, “Antioxidant”, “Inflammation”, “Dental Pain”, “Systematic Review”, “Clinical Trials”, “Orthodontics”, “Herbal Medicine”, and “Phytotherapy”. The search string utilized these terms in combination with Boolean operators to refine the search effectively. For example, the search string was structured as follows: ((“Orthodontic Appliances” OR “Orthodontic Treatment” OR “Orthodontic Patients” OR “Orthodontic Care” OR “Braces” OR “Oral Surgery” OR “Maxillofacial Surgery”) AND (“Natural Products” OR “Natural Remedies” OR “Herbal Medicine”) AND (“Anti-Inflammatory” OR “Oxidative Stress” OR “Inflammation”) AND (“Dental Pain” OR “Pain Reduction”) AND (“Systematic Review” OR “Clinical Trials”)).

Following the Preferred Reporting Items for Systematic reviews and Meta-Analyses (PRISMA) guidelines [19], this protocol was established to ensure clarity, structure, and reproducibility in the review process. The review has been registered with the Open Science Framework (OSF) with the registration number osf.io/rvsqb, underscoring our commitment to a transparent and rigorous systematic review process.

### 2.2. Eligibility Criteria

The inclusion criteria were set as follows: (1) Study population: studies must involve patients undergoing orthodontic treatment without restrictions on age, sex, or ethnicity to cover a diverse demographic; (2) Intervention: research explicitly investigating the use of natural products as anti-inflammatory and antioxidant agents in orthodontic care, with effects on inflammation reduction, pain alleviation, and improvement in treatment compliance and patient satisfaction; (3) Study types: only randomized controlled trials (RCTs) were considered, ensuring the highest level of evidence; (4) Outcome measures: studies must utilize validated assessment tools or well-defined parameters for measuring the outcomes of inflammation and pain management; (5) Language and peer review: only peer-reviewed articles published in English were included to ensure accessibility and quality of the data.

Exclusion criteria were also specified to refine the study selection: (1) Non-human studies: in vitro or animal studies were excluded to focus exclusively on human clinical outcomes; (2) Non-specific interventions: studies that did not specifically investigate natural products as the primary intervention for anti-inflammatory purposes in orthodontic treatment were excluded; (3) Insufficient detail: studies lacking specific, quantifiable outcomes related to the effectiveness and safety of the natural products used, or those missing sufficient detail for a thorough evaluation, were excluded; (4) Observational studies, cohort studies, case-control studies, and quasi-experimental studies were excluded to maintain a focus on RCTs; and (5) non-scientific publications: non-peer-reviewed articles, conference proceedings, theses, dissertations, general reviews, commentaries, and editorials were also excluded to ensure that the review was based on scientifically valid and peer-evaluated sources.

### 2.3. Data Collection Process

**Figure 1:**
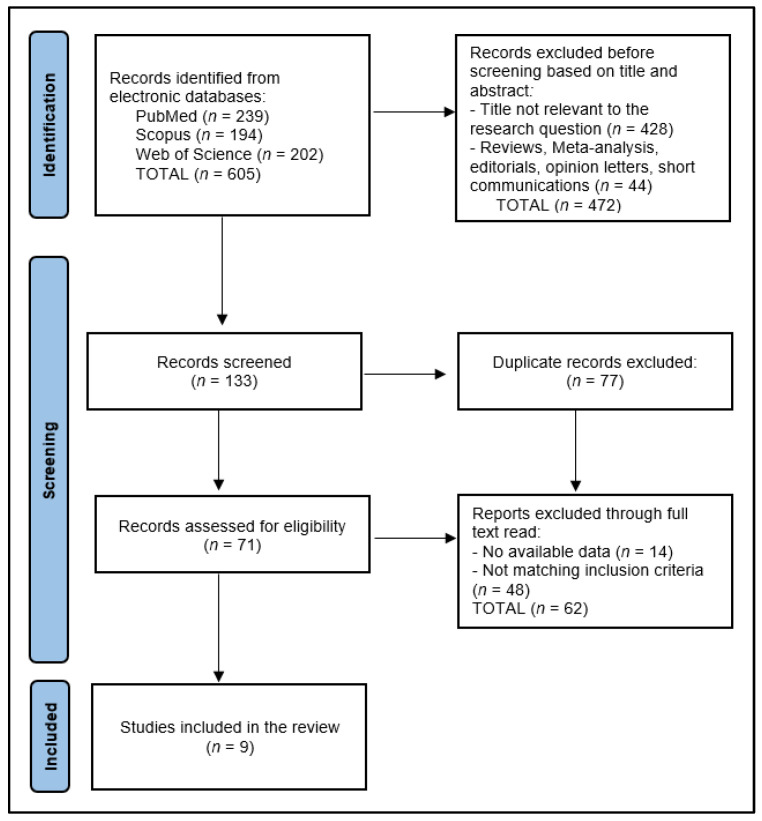
PRISMA flowchart.

Data was processed using Microsoft Excel version 2023 (Microsoft Corporation, Redmond, WA, USA) and graphics were created using Python version 3.9 (Wilmington, DE, USA). The data collection process for this systematic review commenced with the removal of 77 duplicate entries, followed by a rigorous screening of 133 abstracts by two independent reviewers to assess each study’s relevance based on predefined inclusion and exclusion criteria. Of the 71 records assessed for eligibility, 14 were excluded by having no available data or by not matching the inclusion criteria (*n* = 48). Discrepancies between reviewers were resolved through discussion or, if necessary, consultation with a third reviewer to achieve consensus. The initial database search yielded a number of 605 articles, from which 9 relevant studies were identified for inclusion in the final study, as presented in Figure 1.

### 2.4. Risk of Bias and Quality Assessment

For the systematic assessment of study quality and determination of the risk of bias within the included studies, our review employed a dual approach, integrating both qualitative and quantitative evaluation methods. Initially, the quality and risk of bias of clinical trial studies were evaluated using Cochrane’s tool [20]. Discrepancies in quality assessment scores were resolved through discussion or, if necessary, consultation with a third researcher.

## 3. Results

### 3.1. Study Characteristics

The systematic review included a total of nine studies [21,22,23,24,25,26,27,28,29], as summarized in Table 1. These studies were conducted in various countries, including Spain [21,23], the United States [22], India [24,27], Brazil [25,26], Egypt [28], and Iran [29], over a period spanning from 2014 to 2022. Each study employed a randomized clinical trial design, focusing on evaluating the therapeutic properties of natural products in orthodontic treatment. The quality assessment of these studies revealed a mix of medium and high ratings. Specifically, five studies [21,24,25,26,28] were rated as medium quality, indicating satisfactory adherence to standard research methodologies but potentially possessing some limitations in study design or execution. In contrast, four studies [22,23,27,29] received high-quality ratings, suggesting a robust study design, comprehensive methodology, and reliable results that adhere closely to the highest standards of clinical research. This diversity in study quality and geographic distribution underscores the global interest in natural therapies within orthodontic treatment, reflecting a broad academic and clinical investment in exploring alternative and supportive treatment modalities.

### 3.2. Population Characteristics

These studies incorporated a total of 358 participants in the intervention groups, exploring the efficacy of natural products in orthodontic treatment. The age of participants varied widely across the studies. López-Mateos et al. [21] and Kamath et al. [24] reported older intervention groups with mean ages of 32.2 years and 22.5 years, respectively, contrasting sharply with the predominantly adolescent cohorts in studies by Furtado Júnior et al. [25] and Atwa et al. [28], who reported age ranges of 12–16 years and 12–18 years, respectively. The broadest age range was observed in the study by Goes et al. [26], where participants ranged from 10 to 40 years, with a mean age of 28.8 years, illustrating the inclusion of both adolescent and adult orthodontic patients.

The gender distribution also showed considerable variability. The studies by López-Mateos et al. [21] and Kamath et al. [24] demonstrated a higher female participation in the intervention groups, with 85.4% and 60.0%, respectively, reflecting a common trend in orthodontic studies where females often represent a larger proportion of the sample. In contrast, studies by Martin et al. [22] and Yeturu et al. [27] maintained a balanced gender ratio, each with an even 50% distribution, indicating a deliberate design to eliminate gender as a confounding factor.

Control groups varied across studies, providing a spectrum of comparators that enriched the review’s insights into the efficacy of natural products. For example, López-Mateos et al. [21] used patients treated with brackets as a control group, whereas Goes et al. [26] included both placebo and chlorhexidine-treated patients, offering a robust basis for comparison against the natural product interventions (Table 2).

### 3.3. Clinical Trial Assessment

Each study varied in the substance tested, the application method, and the frequency and duration of follow-up, providing a comprehensive view of the experimental approaches in the use of natural products for orthodontic care. López-Mateos et al. [21] assessed the impact of orthodontic aligners on saliva’s oxidative stress markers, using advanced procedures to collect and analyze saliva samples at baseline and subsequent intervals up to 90 days. They utilized both Invisalign^®^ and Damon System^®^ brackets, tracking changes over three months, which is significant for understanding the long-term biochemical impacts of orthodontic appliances on saliva.

Martin et al. [22] focused on an antioxidant essential oil gel, examining its effect on gingival health by applying the gel twice daily, with evaluations every 4–6 weeks. Their use of both active and placebo gels provided a controlled environment to assess the gel’s efficacy over a standard follow-up period, ensuring rigorous testing of the product’s therapeutic properties. Leiva-Cala et al. [23] and Kamath et al. [24] both utilized Aloe vera in different forms (gel and mouthwash) compared directly against chlorhexidine, a standard in periodontal treatment. Their studies included detailed follow-up assessments to monitor immediate and short-term changes in oral health parameters, essential for evaluating the effectiveness of natural products against established chemical treatments.

Furtado Júnior et al. [25] and Goes et al. [26] both incorporated natural ingredients (Brazilian red propolis and *Matricaria chamomilla* L., respectively) into daily oral hygiene routines, with Furtado Júnior et al. assessing outcomes after 28 days and Goes et al. after 15 days. These studies provided insight into the practical application and benefits of incorporating natural products into everyday oral care. Atwa et al. [28] utilized a unique approach by integrating dietary natural products (honey) directly compared with traditional dietary sugars (sucrose and sorbitol) to evaluate their effects on plaque pH over a concise timeframe of 30 min, offering immediate biochemical results. Golshah et al. [29] extended their observation period up to 8 weeks to monitor the effectiveness of an emulgel containing 2% resveratrol on gingival health, providing substantial evidence of the long-term benefits of resveratrol in managing gingivitis in orthodontic patients (Table 3).

### 3.4. Assessment of Outcomes

Several studies documented significant outcomes. López-Mateos et al. [21] observed a notable increase in Advanced Oxidative Protein Product (AOPP) levels from 47.1 μM at baseline to 79.5 μM at 90 days in aligners and from 57.8 μM to 80.2 μM in self-ligating brackets. This suggests that both treatments could potentially elevate oxidative stress markers over time. However, changes in myeloperoxidase (MPO) and total antioxidant capacity (TAC) were not statistically significant, indicating a selective impact on specific oxidative stress markers. Martin et al. [22] reported an initial significant decrease in Bleeding on Probing (BOP) from baseline (T1) to the subsequent time points (T2) by 13.6 in the treatment group compared to a decrease of 3.0 in the placebo group. The Gingival Index (GI) slightly improved by 0.02 in the treatment group compared to a worsening of 0.06 in the placebo, suggesting that the topical gel initially reduces inflammation markers effectively.

Leiva-Cala et al. [23] found that Aloe vera gel significantly reduced inflammation by 41.4% compared to chlorhexidine, which underscores the potential of Aloe vera as a more effective and less adverse option for managing oral inflammation in orthodontic patients. Kamath et al. [24] demonstrated a decrease in the Gingival Index (GI) by 0.64 in the Aloe vera group versus 0.54 in the chlorhexidine group over the study duration. The reduction in bleeding on probing (BOP) was also more pronounced in the Aloe vera group (23.7) compared to chlorhexidine (29.2), reinforcing the viability of Aloe vera as an alternative treatment.

Furtado Júnior et al. [25] showed significant reductions in the Gingival Bleeding Index (GBI) and microbial counts with the use of fluoride dentifrice containing Brazilian red propolis. The GBI decreased by 17.35 in the propolis group compared to 11.11 in the control, and reductions in Gram-negative bacteria and S. mutans were also significantly more pronounced in the propolis group, indicating improved clinical and microbiological activity. Goes et al. [26] observed significant reductions in the Visible Plaque Index (VPI) by 25.6% in the *Matricaria chamomilla* L. (MTC) group and 39.9% in the chlorhexidine group compared to the placebo, alongside notable decreases in the Gingival Bleeding Index (GBI) by 29.9% and 32.0%, respectively, emphasizing the effectiveness of MTC mouthwash as a herbal alternative with fewer side effects.

**Figure 2:**
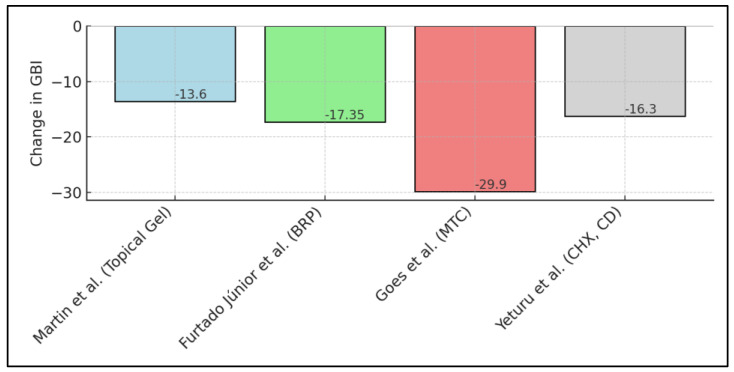
Gingival bleeding index changes (before and after intervention) across different natural compounds: BRP—Brazilian red propolis; MTC—*Matricaria chamomile*; CHX—chlorhexidine; CD—chlorine dioxide. Data is from [22,25,26,27].

Yeturu et al. [27] documented reductions in plaque and gingival scores with different treatments: Aloe vera reduced plaque scores by 20.38% and gingival scores by 9.88%, while chlorhexidine reduced plaque by 31.59% and gingival scores by 16.30%, with chlorine dioxide also showing substantial efficacy. Atwa et al. [28] highlighted significant pH changes with honey, which reduced from 6.85 to 5.86 before recovering to 6.84, indicating honey’s rapid pH modulation ability. Furthermore, bacterial counts significantly decreased, with Streptococcus mutans reducing from 255.6 to 104.4 CFU, showcasing honey’s strong antibacterial properties beneficial for orthodontic patients. Golshah et al. [29] reported a significant reduction in the Gingival Index (GI) from 1.00 to 0.77 over 8 weeks in the experimental group using an emulgel containing 2% resveratrol, highlighting its potential for managing gingivitis effectively in orthodontic treatment settings, as presented in Table 4 and Figure 2.

## 4. Discussion

### 4.1. Summary of Evidence

The systematic review underscores the nuanced therapeutic properties of natural products in orthodontic treatment. López-Mateos et al. [21] reported significant increases in oxidative stress markers with both aligners and brackets over 90 days, highlighting the potential long-term biochemical impacts of orthodontic appliances on oral health. This study suggests a pivotal need to explore further the implications of oxidative stress in orthodontic treatments and its management with potentially antioxidative natural interventions.

Martin et al. [22], focusing on the therapeutic effects of an antioxidant essential oil gel, demonstrated short-term effectiveness in reducing gingival inflammation. This suggests that while natural products can initially modulate inflammatory responses effectively, their sustained impact post-discontinuation remains uncertain. This transient nature of effectiveness prompts a discussion on the necessity for ongoing treatment or perhaps the integration of such natural products into regular dental care routines to maintain their beneficial outcomes.

On the other hand, Leiva-Cala et al. [23] and Kamath et al. [24] utilized Aloe vera against chlorhexidine, a standard antiseptic, to underline the potential of natural products as viable alternatives for chemical treatments. Their findings, which favor Aloe vera, challenge the traditional reliance on chemical antiseptics in orthodontics, advocating for a paradigm shift towards integrating bioactive, natural substances that exhibit comparable efficacy with fewer side effects [30,31,32].

Furtado Júnior et al. [25] and Goes et al. [26] further contribute to this narrative by demonstrating the clinical and microbiological benefits of integrating natural ingredients like Brazilian red propolis and *Matricaria chamomilla* L. into daily oral hygiene. These studies underscore the significant role that such ingredients can play in enhancing oral health parameters, thus supporting their incorporation into standard oral care products.

In the analysis of natural remedies in orthodontic practice, several studies have attempted to identify effective treatments that mitigate complications associated with fixed appliances. For example, the study by Papadopoulou et al. [33] focused on the impact of organic, unprocessed products like Aloe vera, honey, and chamomile mouthwash on controlling gingivitis among orthodontic patients. They found significant reductions in plaque and gingival bleeding, attributing these benefits to the antimicrobial and anti-inflammatory properties of the natural products used. On the other hand, Inchingolo et al. [34] explored the role of antioxidants such as resveratrol and green tea in countering oxidative stress and reactive oxygen species production induced by orthodontic treatment. These studies suggest that these natural antioxidants and dietary supplements can help restore physiological balance, reduce inflammation levels, and prevent damage to dental and periodontal tissues, highlighting a preventive strategy against long-term complications in orthodontic care [35,36,37,38,39].

In examining the impact of orthodontic materials on oxidative stress, other studies provide valuable insights through contrasting methodologies and findings [34,40,41,42]. However, the non-clinical trial study design did not allow for the inclusion of the current systematic review. Portelli et al.’s study [43] measured salivary antioxidants in patients using self-ligating multibracket vestibular orthodontic appliances and found that antioxidant levels slightly decreased from 2971 mEq/L at baseline to 2909 mEq/L after five weeks, then increased to 3332 mEq/L after ten weeks, without statistically significant changes, suggesting minimal impact on oxidative stress. In contrast, Vito Kovac et al. [44] investigated the cytotoxicity and oxidative stress potential of orthodontic alloys in yeast cells, revealing that stainless steel and cobalt-chromium at 1000 µM concentration were cytotoxic, with significant oxidative damage observed even at 100 µM for the cobalt-chromium alloy, highlighting the potential for these materials to induce oxidative stress depending on their composition and exposure levels.

Other studies examined oxidative stress in patients with fixed orthodontic appliances, revealing complex biological responses. Buczko et al. [45] observed significant increases in salivary markers such as tiobarbituric acid reactive substances and total oxidant status shortly after appliance placement, with tiobarbituric acid reactive substances rising one week post-treatment and total protein concentration increasing significantly at twenty-four weeks. On the other hand, Angeles-Estrada et al. [46] reported increases in the oxidative stress marker 8-hydroxy-2′deoxyguanosine and significant changes in antioxidant enzymes, with superoxide dismutase levels increasing by 2.5 times after six months and catalase tripling after six months, then returning to baseline by nine months.

Ozcan et al. [47] and Guler et al. [48] conducted focused studies on the biochemical impacts of fixed orthodontic appliances. Ozcan et al. examined the levels of interleukin-1 beta, tumor necrosis factor alpha, malondialdehyde, nitric oxide, and 8-hydroxydeoxyguanosine in saliva and gingival crevicular fluid over six months. They found that except for interleukin-1 beta in gingival crevicular fluid, which significantly increased by the sixth month, other markers did not change significantly, suggesting minimal systemic oxidative damage. Conversely, Guler et al. evaluated the impact of different orthodontic composites on salivary total oxidant status, total antioxidant status, and 8-hydroxy-2′-deoxyguanosine in children, finding that while total antioxidant status significantly decreased over time in composites like Kurasper F and GrenGloo, 8-hydroxy-2′-deoxyguanosine initially decreased and then significantly increased by the third month, indicating dynamic changes in oxidative stress related to material composition and treatment duration.

Other studies investigated the effectiveness of different dental hygiene products with natural components in managing oral health in the context of orthodontic treatment. Santamaria et al. [49] studied the antimicrobial effects of Melaleuca alternifolia essential oil gel compared to Colgate Total toothpaste. Over a four-week period involving a crossover design, they found that the melaleuca gel significantly reduced dental biofilm and bacterial colonies more effectively than Colgate Total. However, in terms of sensory attributes such as flavor and initial taste sensation, Colgate Total was preferred by volunteers (*p* < 0.05). Conversely, Masou et al. [50] conducted a pilot study on the use of xylitol, delivered through chewing gum and dissolvable tablets, to assess its impact on dental plaque and bacterial levels in patients with fixed orthodontic appliances. Their study, which also included oral hygiene instruction and fluoride treatments, found no significant difference in plaque scores or bacterial counts between the xylitol groups and a control group over a 12-month period, suggesting that xylitol did not provide additional clinical or bacterial benefits in this context. However, the study design did not allow for inclusion and detailed analysis in the current systematic review because it was not a clinical trial.

Aloe vera has been extensively studied for its therapeutic effects in oral health, being rich in bioactive compounds such as glycoproteins, which help to reduce pain and inflammation, and polysaccharides, which promote healing and moisturize the tissues [51]. Many studies have demonstrated that Aloe vera can significantly reduce gingival inflammation and bleeding, which are common complications in orthodontic treatments. Its antimicrobial properties, attributed to compounds such as barbaloin and acemannan, also make it effective against common oral pathogens, enhancing its suitability for dental applications [52,53].

A formulation based on a fluoride dentifrice enhanced with Brazilian red propolis is another notable natural product in orthodontic care [54]. Brazilian red propolis contains unique compounds such as formononetin, biochanin A, and medicarpin, which are known for their anti-inflammatory and antimicrobial activities. These properties help reduce gingival inflammation and control microbial populations in the oral cavity, effectively combating dental plaque and periodontal pathogens. Another study showed that the addition of fluoride dentifrice enhances its ability to prevent dental caries, making this formulation effective for maintaining oral hygiene in orthodontic and orthognathic patients [55].

*Matricaria chamomilla* L., commonly known as chamomile, was another natural compound that was tested in a clinical trial and found to be effective. It can be utilized in the form of a mouthwash and has shown promising results in reducing plaque accumulation and gingival bleeding [56]. Chamomile contains bisabolol, chamazulene, and flavonoids, which are compounds with potent anti-inflammatory and antiseptic properties [57]. These components help soothe irritated mucous membranes and reduce bacterial activity, thus preventing inflammation and plaque buildup. Its use as a mouthwash in orthodontic patients provides a soothing, therapeutic effect, complementing traditional mechanical plaque control methods [58].

Overall, the most recent studies offer significant insights into the use of natural products and novel materials in dentistry, emphasizing their efficacy and potential applications. Eskandari et al. [59] demonstrated that elastomeric ligature ties coated with kombucha-derived bacterial nanocellulose maintain their mechanical properties while providing sustained antibacterial activity, which is crucial for preventing carious lesions in orthodontic patients. Another study [60] highlighted the broad therapeutic potential of Cissus extracts in dentistry, noting their antibacterial, anti-inflammatory, and tissue-regenerative properties, which could modernize dental practices. It is also worth mentioning the latest views on phytotherapy, which revealed that herbal treatments, such as mouthwashes, significantly enhance oral health-related quality of life, are cost-effective, and improve healthcare access [61]. Lastly, Pedrinha’s study on propolis and copaiba oil-resin showed these natural antimicrobials promote proliferation in human periodontal ligament fibroblasts without cytotoxic effects, suggesting their safe use in endodontic and other medical applications to manage inflammation and infection [62]. These studies collectively underline the transformative potential of natural and innovative materials in enhancing dental care outcomes.

### 4.2. Limitations

While this systematic review employs a comprehensive approach to assess the efficacy of natural products in orthodontic care, several limitations are inherent to its design and scope. Firstly, the variability in natural products and their application methods poses a challenge in drawing generalized conclusions, as different studies may use distinct formulations, concentrations, and application protocols, potentially leading to heterogeneous results. Secondly, the review’s reliance on randomized controlled trials published its breadth, potentially excluding relevant studies conducted in other languages or significant findings published in other types of research articles. Moreover, the inclusion criteria might omit studies involving broader demographic characteristics or varying orthodontic conditions that could influence the generalizability of the findings. Additionally, the specific focus on anti-inflammatory, antibacterial, and antioxidant effects may overlook other relevant biological or clinical impacts of natural products. Lastly, the extraction and synthesis of data from different studies require assumptions and interpretations that may introduce bias, particularly when comparing studies with diverse methodologies or outcome measures.

## 5. Conclusions

This systematic review confirms that natural products significantly enhance orthodontic treatment outcomes by reducing inflammation and bacterial levels, attributable to their active chemical compounds. For instance, Aloe vera’s effectiveness in reducing gingival inflammation may be linked to compounds such as aloin and barbaloin, which exhibit anti-inflammatory and antibacterial properties. Similarly, the antibacterial action of honey, particularly against Streptococcus mutans, can be attributed to its natural peroxide content. Resveratrol’s reduction in gingival indices is supported by its antioxidant properties that modulate inflammatory pathways. These findings suggest that natural products not only serve as viable alternatives to traditional treatments but also offer a potential for integration into routine orthodontic care, promising fewer side effects and enhanced patient outcomes. It is, therefore, recommended that natural products such as Aloe vera, resveratrol, Brazilian red propolis, and *Matricaria chamomile* should be introduced in routine practice for orthodontic and orthognathic patients in association with anti-inflammatory and anti-bacterial agents such as chlorhexidine or alike. Further research is recommended to standardize these treatments for broader clinical applications.

## Figures and Tables

**Table 1:** Study characteristics.

Study and Author	Country	Study Year	Study Design	Study Quality
1 [21] López-Mateos et al.	Spain	2022	Randomized clinical trial	Medium
2 [22] Martin et al.	United States	2016	Randomized clinical trial	High
3 [23] Leiva-Cala et al.	Spain	2019	Randomized clinical trial	High
4 [24] Kamath al.	India	2022	Randomized clinical trial	Medium
5 [25] Furtado Júnior et al.	Brazil	2020	Randomized clinical trial	Medium
6 [26] Goes et al.	Brazil	2016	Randomized clinical trial	Medium
7 [27] Yeturu et al.	India	2016	Randomized clinical trial	Medium
8 [28] Atwa et al.	Egypt	2014	Randomized clinical trial	Medium
9 [29] Golshah et al.	Iran	2021	Randomized clinical trial	High

**Table 2:** Characteristics of study groups.

Study Number	Sample Size (Intervention Group) *	Mean Age/Age Range	Gender Distribution (Women)	Control Group(s)
1 [21] López-Mateos et al.	Total: 67 patients, of which 48 had clear aligners	Intervention group (aligners): 32.2 years Control group (brackets): 29.3 years	Intervention group (aligners): 85.4% Control group (brackets): 78.9%	19 patients with brackets
2 [22] Martin et al.	Total: 32 patients, of which 16 patients were treated with antioxidant essential oil gel	Intervention group: 15.9 years Control group: 15.1 years	Intervention group: 50% Control group: 50%	16 patients in placebo group treated with a water-based gel
3 [23] Leiva-Cala et al.	70 patients treated with Aloe vera gel	12 years and older, mean: 26.1 years	63.6%	70 patients treated with 0.12% chlorhexidine gel
4 [24] Kamath al.	Total: 30 patients, of which 15 patients had fixed orthodontic appliances treated with Aloe vera mouthwash	Intervention group: 22.5 years Control group: 22.7 years	Intervention group: 60.0% Control group: 53.3%	15 patients with fixed orthodontic appliances treated with 0.2% chlorhexidine mouthwash
5 [25] Furtado Júnior et al.	Total: 52 patients, of which 46 participants were treated with fluoride dentifrice and Brazilian red propolis	12–16 years	50.0%	46 participants treated with regular fluoride dentifrice
6 [26] Goes et al.	Total: 30 participants with fixed orthodontic appliances, of which 10 were treated with MTC	10–40 years, mean: 28.8 years	86.7%	10 patients in the placebo group, 10 patients treated with 0.12% chlorhexidine mouthwash
7 [27] Yeturu et al.	Total: 90 participants undergoing fixed orthodontic treatment, of which 30 were treated with Aloe vera, 30 with chlorhexidine, and 30 with chlorine dioxide	Mean age: 21.65 years	50.0%	NR
8 [28] Atwa et al.	Total: 20 orthodontic patients treated with honey and 10% sucrose or 10% sorbitol	12–18 years	100%	Sucrose 10% (positive control), sorbitol 10% (negative control)
9 [29] Golshah et al.	Total: 46 orthodontic patients, of which 23 were treated with emulgel	12–25 years	Experimental: 57.9% female, Placebo: 57.9% female, Control: 57.9% female	23 patients in the placebo group that used no product

NR—Not Reported; * The intervention group was defined as patients that have received the natural product from the experiment vs. the control group (patients with the same dental procedures that have not received the natural dental product of the study); MTC—Matricaria chamomile.

**Table 3:** Clinical trial assessment.

Study Number	Substance	Measurement/Dose/Administration	Follow-Up	Materials Used
1 [21] López-Mateos et al.	Advanced oxidative protein product of saliva; total antioxidant capacity; myeloperoxidase activity	Saliva collection: between 08:30 and 09:00 after fasting and chewing paraffin for 5 min; initial secretion discarded, followed by 5 min of collection, then frozen at −80 °C and centrifuged at 3000 rpm for 20 min	At baseline before starting treatment, and then at 30 and 90 days before placing the next apparatus in the sequence	Aligners: Invisalign^®^ system (Align Technology, San Jose, CA, USA), worn 22 h per day Brackets: Damon System^®^ 0.22″ self-ligating brackets Q passive self-ligating brackets (Ormco Corporation, Orange, CA, USA) combined with Damon Optimal Force Copper Ni-Ti^®^ 0.014″ archwires
2 [22] Martin et al.	Antioxidant essential oil gel (containing menthol, thymol, ferulic acid, phloretin)	Applied twice daily after brushing to the gingiva, followed by a 30-min non-rinse period	Periodic assessments at T1, T2, and T3 (approximately every 4–6 weeks)	Placebo gel or active gel (AO ProVantage Dental Gel, Periosciences, Dallas, TX, USA) applied to the gingiva of patients with fixed orthodontic appliances
3 [23] Leiva-Cala et al.	Aloe vera gel vs. 0.12% chlorhexidine gel	Aloe vera or chlorhexidine gel applied twice daily after tooth brushing, massaging for 15 min	Periodic assessments; initial and 1-month follow-up	80% of Aloe vera combined with carbopol, a cross-linked acrylic acid hydrophilic polymer, and ascorbic acid. The CHX formulation used is commercially available as 0.12% Lacer Bioadhesive Gel (Lacer S.A., Barcelona, Spain)
4 [24] Kamath al.	Aloe vera mouthwash vs. 0.2% chlorhexidine mouthwash	Aloe vera or chlorhexidine mouthwash used twice daily, 10 mL for 1 min	Assessments at baseline, 21 days, and 35 days	Aloe vera mouthwash consisted of pure Aloe vera juice (Aloe vera Juice, Patanjali Ayurveda Ltd., Kochi, India), derived from the pulp of the leaf. Composition of each 10 mL: 99.6% (*w*/*v*) Aloe vera juice; 0.02% (*w*/*v*) citric acid crystal; 0.02% (*w*/*v*) sodium benzoate crystal (preservative); and 0.2% Chlohex plus mouthwash
5 [25] Furtado Júnior et al.	Fluoride dentifrice with and without Brazilian red propolis	Dentifrice used twice daily with standard brushing	Baseline and after 28 days	First, 150 g of the red propolis extract was taken and extracted with 1 L of cereal alcohol of 96° graduation and then diluted to a concentration of 1%. BRP extract at 1% concentration (previously studied antimicrobial concentration) was incorporated into the fluoridated dentifrice (1500 ppm).
6 [26] Goes et al.	1% *Matricaria chamomilla* L. mouthwash	Used twice daily, 15 mL for 1 min	Baseline and after 15 days	The mouthwash was prepared from *Matricaria chamomilla* L. extract; placebo and 0.12% chlorhexidine used as controls
7 [27] Yeturu et al.	*Aloe vera*, chlorine dioxide, chlorhexidine	Mouth rinse used twice daily; 10 mL for 1 min	Baseline and after 15 days	NR
8 [28] Atwa et al.	Honey	Patients were then asked to chew and ingest 10 g of pure undiluted honey in 2 min or rinse with 15 mL of 10% sucrose or sorbitol solutions (positive and negative controls, respectively) for 1 min	pH measured prior to baseline and at 2, 5, 10, 20, and 30 min after treatment.	Honey, sucrose, and sorbitol
9 [29] Golshah et al.	emulgel containing 2% resveratrol	5 mL applied and massaged onto the gums nightly for 30 s	Baseline, 4 weeks, and 8 weeks	Emulgel containing 2% resveratrol; identical placebo emulgel without resveratrol; no product for control

NR—not reported; BRP—Brazilian red propolis; CHX—chlorhexidine.

**Table 4:** Assessment of outcomes.

Study Number	Therapeutic Effects	Other Outcomes	Interpretation
1 [21] López-Mateos et al.	AOPP (μM)–Aligners: T0 (47.1)–T90 (79.5) *. Self-ligating brackets: T0 (57.8)–T90 (80.2). MPO (mUl/mL)–Aligners: T0 (8.4) to T30 (13.9) and T90 (12.7). Self-ligating brackets: T0 (14.3)–T30 (9), and T90 (12). TAC (μM)–Aligners: T0 (50)–T90 (51.6). Self-ligating brackets: T0 (49.1)–T30 (53.5), and T90 (49.8).	NR	Overall, aligners showed a significant increase in AOPP over the 90 days, particularly in the later stages, whereas changes in MPO and TAC were not significant for either orthodontic technique.
2 [22] Martin et al.	BOP: T1 − T2 = −13.6 in treatment group vs. −3.0 in placebo group *; GI: T1 − T3 = −0.02 in treatment group vs. +0.06 in placebo group *; PD: T1 − T2 = −0.03 in treatment group vs. +0.05 in placebo group *	The plaque index showed no significant differences between the treatment and control groups at any point during the study period.	Topical gel effectively reduced inflammation markers initially; however, the effects were not sustained post-discontinuation of the gel application.
3 [23] Leiva-Cala et al.	Inflammation: 10.0% with Aloe vera vs. 51.4% with chlorhexidine *	Ulceration: 5.7% with Aloe vera vs. 81.4% with chlorhexidine *	Aloe vera gel demonstrated effective prevention of oral inflammation in orthodontic patients, significantly outperforming chlorhexidine gel without adverse effects
4 [24] Kamath al.	GI: T1 − T2 = −0.64 in the Aloe vera group vs. −0.54 in the chlorhexidine group *; BOP: T1 − T2 = −23.7 in the Aloe vera group vs. −29.2 in the chlorhexidine group *	PI (Plaque Index): T1 − T2 = −0.56 in the Aloe vera group vs. −1.07 in the chlorhexidine group *	Both mouthwashes effectively managed plaque and gingivitis in orthodontic patients, suggesting Aloe vera as a viable alternative to chlorhexidine with fewer side effects.
5 [25] Furtado Júnior et al.	GBI: T1 − T2 = −11.11 in the control group vs. −17.35 in the BRP group; * Gram-negative bacteria: T1 − T2 = +0.45 in the control group vs. −0.61 in the BRP group; * *S. mutans*: T1 − T2 = −0.06 in the control group vs. −0.33 in the BRP group.*	CFU counts for *S. mutans* and Gram-negative bacteria were significantly lower in the BRP group compared to the control group after 28 days	BRP dentifrice demonstrated better clinical and microbiological activity after 28 days, significantly reducing oral bacteria levels and improving gingival health compared to regular fluoride dentifrice
6 [26] Goes et al.	VPI: Significant decrease in the MTC group (−25.6%) and the chlorhexidine group (−39.9%) compared with placebo; GBI: Significant decrease in the MTC group (−29.9%) and the chlorhexidine group (−32.0%) compared with placebo	Improvement in oral hygiene and reduction of gingival inflammation	MTC mouthwash significantly reduced plaque accumulation and gingival bleeding, offering an effective herbal alternative to chlorhexidine with fewer side effects
7 [27] Yeturu et al.	Plaque score reduction: Aloe vera (−20.38%), chlorhexidine (−31.59%), chlorine dioxide (−30.29%); Gingival score reduction: Aloe vera (−9.88%), chlorhexidine (−16.30%), and chlorine dioxide (−12.22%)	NR	Chlorhexidine had the highest effectiveness in reducing plaque and gingival scores, with chlorine dioxide also being a cost-effective alternative
8 [28] Atwa et al.	pH: Significant pH changes observed. Honey (6.85 to 5.86, then recovery to 6.84); sucrose (6.82 to 5.28); sorbitol stable around 6.86. Bacterial counts: *Streptococcus mutans* reduced from 255.6 to 104.4 CFU, *Lactobacilli* from 100.2 to 42.2 CFU, and *P. gingivalis* from 56.4 to 42 CFU with honey.	NR	Honey demonstrated a rapid recovery in plaque pH and significantly reduced bacterial counts compared to sucrose and sorbitol, suggesting strong antibacterial effects beneficial for orthodontic patients.
9 [29] Golshah et al.	GI: Significant reduction from T0 to T2 in the experimental group (1.00 to 0.77); BOP: No significant change; PPD: decrease from T0 to T2 in the experimental group (2.23 to 1.29); HI: significant reduction from T0 to T2 in the experimental group (1.52 to 0.68) *	NR	Emulgel containing 2% resveratrol significantly improved clinical parameters of gingival health over 8 weeks, reducing GBI, HI, and PD effectively compared to placebo and control groups. Demonstrates potential for use in managing gingivitis in orthodontic patients.

*—Statistically significant differences (*p*-value < 0.05); AOPP—Advanced oxidative protein product of saliva; BOP—Bleeding on probing; CFU—Colony Forming Units; CHX—chlorhexidine; GBI—gingival bleeding index; HI—Healing Index; MPO—myeloperoxidase; MTC—Matricaria chamomile; NR—not reported; PD—probing depth; TAC—total antioxidant capacity; T—time of measurement; VPI—Visible Plaque Index; BRP—Brazilian red propolis.
